# P-1179. Investigations of carbapenemase-producing bacteria among children in U.S. healthcare settings: review of CDC consultations, 2015–2023

**DOI:** 10.1093/ofid/ofae631.1365

**Published:** 2025-01-29

**Authors:** Mohsin Ali, Danielle A Rankin, Christopher Prestel, Maroya S Walters

**Affiliations:** CDC, Decatur, Georgia; Centers for Disease Control and Prevention, Atlanta, Georgia; Centers for Disease Control and Prevention, Atlanta, Georgia; Centers for Disease Control and Prevention, Atlanta, Georgia

## Abstract

**Background:**

The US Centers for Disease Control and Prevention (CDC) provides technical assistance (consultations) to health departments responding to single cases and clusters of carbapenemase-producing organisms (CPOs). We aimed to characterize consultations involving pediatric patients.

**Figure 1.**
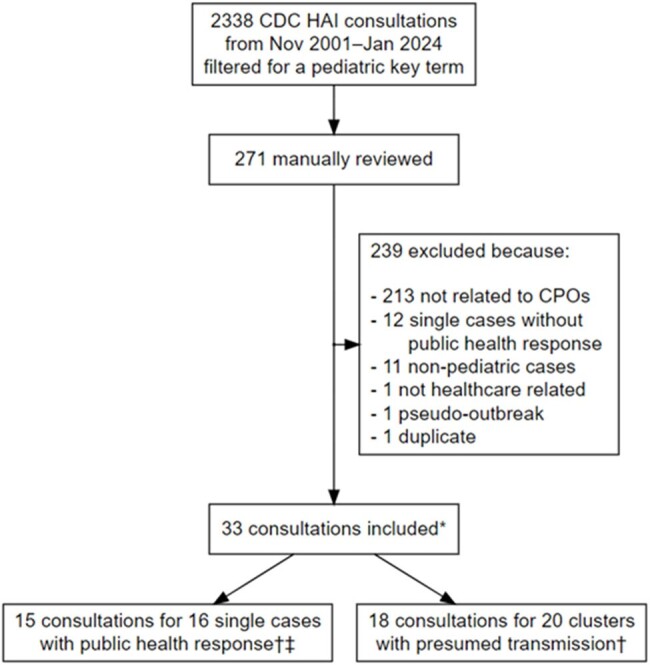
Flow diagram of screening of CDC consultations for pediatric CPO cases requiring public health responses or clusters with suspected or confirmed transmission. Abbreviations: AR, antimicrobial resistance; CPO, carbapenemase-producing organism; HAI, healthcare-associated infection. *Includes one additional recent consultation not yet added to database at time of query. †One consultation involved two unrelated single cases and another consultation included 3 subclusters. ‡Applicable public-health response components were contact screening, site visit/infection control assessment, and/or environmental sampling.

**Methods:**

We searched CDC databases of healthcare-associated infection (HAI) consultations (November 2001 to January 25, 2024) using pediatric key-word patterns. We reviewed this subset for CPO investigation consultations with at least one pediatric case (age < 18 years with a CPO-positive clinical or screening specimen) or involving a pediatric healthcare facility. We classified CPO consultations as either (a) a cluster with likely transmission or (b) a single case with a public-health response (Figure 1). We abstracted and descriptively analyzed consultation characteristics regarding microbiology, healthcare setting, index patient characteristics, public-health response, and likely transmission mechanisms.

**Figure 2.**
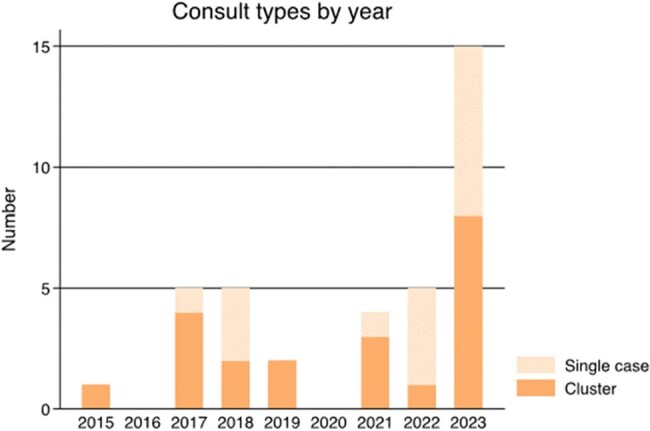
Number of CDC consultations for CPOs by year among children in U.S. healthcare settings, 2015–2023.

**Results:**

Among 2338 HAI consultations, 271 (12%) related to pediatric cases or facilities were reviewed (Figure 1). Thirty-three consultations from 2015–2023 across 20 states were included, which comprised 16 single cases and 20 clusters (Figure 2). Eight (40%) clusters only involved pediatric cases and the remainder included both pediatric and adult cases. Most commonly, for 17 (47%) index cases, Enterobacterales was identified in a clinical specimen from an acute-care hospital patient in an intensive care unit (e.g., burn unit; Table 1). The most common carbapenemases were NDM (53%), KPC (28%), and VIM (22%). Among the 16 clusters with available information, the most common transmission mechanisms were gaps in core infection control practices (9, 56%) and premise plumbing (3, 19%). Overall, 9 (56%) single cases and 6 (30%) clusters had history of international healthcare or travel for the index case-patient.

**Table 1.**
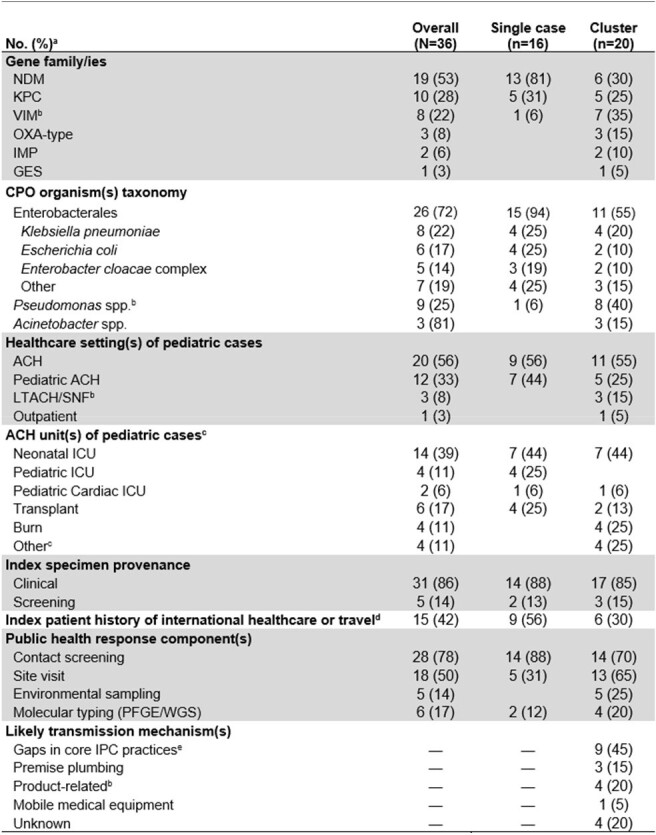
Microbiologic, healthcare setting, index patient, and public health response characteristics of consultations of CPO among children in U.S. healthcare settings, by consultation type, 2015–2023

Abbreviations: ACH, acute care hospital; CPO, carbapenamase-producing organism; ICU, intensive care unit; IPC, infection prevention and control; LTACH, long-term acute-care hospital; PFGE, pulse field gel electrophoresis; SNF, skilled nursing facility; WGS, whole genome sequencing.

a Proportions may not add to 100% as values may not be mutually exclusive per consultation and/or rounding error.

b Three clusters of VIM–P. aeruginosa in different facilities were part of multistate outbreak linked to artificial tears.

c Denominator for clusters is 16 ACHs. “Other” includes one each of surgery, pediatrics, medicine, and adult ICU units.

d Includes three single cases and one cluster where index case was a US-born patient in a neonatal ICU whose mother had history of international healthcare or travel.

e Includes hand hygiene, Transmission-Based Precautions, and cleaning and disinfection of high-touch surfaces in patient rooms.

**Conclusion:**

Review of CDC consultations for CPOs in pediatric patients identified multiple cases and clusters among high-risk inpatient units. Links to healthcare outside the U.S. were common. Admission screening, particularly on high-risk units, and adherence to core infection control practices may prevent future outbreaks.

**Disclosures:**

**All Authors**: No reported disclosures

